# 

**Published:** 1896-08

**Authors:** 


					﻿CONTENTS
ORIGINAL ARTICLES—
Glaucoma in relation to General Practice. By Alex. W. Stirl-
ing, M. D., Atlanta, Ga................................	373
Some Injuries of the Eyeball. By S. Latimer Phillips, M. D.,
Savannah, Ga............................................ 386
SELECTIONS AND ABSTRACTS—
Sleeplessness........................................... 390
Shall Opticians Attempt to Fit Glasses ?................ 392
On the Preparation of Anatomical Material............... 393
Modern Treatment of Eclampsia, Considered from a Medical
Standpoint...........................................  394
Epidemic Typhoid Fever................................   406
EDITORIAL—
When Shall Gonorrhoeries Marry?............... 412
NOTES—	413
BOOK REVIEWS—	415
SPECIAL NOTES—	416
Prescri ption s................................... 424
				

